# The Study on the Capacitive Properties of Tungsten Oxide/Graphene Composites Prepared by Ultrasonic Spray Deposition

**DOI:** 10.1021/acsomega.5c10930

**Published:** 2026-01-26

**Authors:** Chi-Ping Li, Chien-Chung Su

**Affiliations:** Department of Chemical Engineering, National United University, No. 2, Lienda, Maioli 360302, Taiwan

## Abstract

The fabrication of tungsten oxide/graphene thin films on fluorine-doped tin oxide (FTO) glass substrates using ultrasonic spray deposition technology is presented, and their potential application as supercapacitor electrode materials is explored. Ultrasonic spray deposition provides precise control over film thickness, low fabrication cost, scalability for large-area coating, and compatibility with green solvents, making it a sustainable thin-film deposition method. A template-assisted sol–gel chemistry was employed to synthesize mesoporous tungsten oxide films with high specific surface area, enabling enhanced charge adsorption/desorption and improved electrochemical performance. To further enhance the ionic conductivity, a graphene interlayer was introduced between the WO_3_ film and the FTO substrate, forming a composite electrode structure. Electrochemical characterization using cyclic voltammetry (CV), galvanostatic charge–discharge (GCD), and electrochemical impedance spectroscopy (EIS) revealed superior performance of the WO_3_/graphene electrode, achieving a specific capacitance of 161.68 F g^–1^ at a 5 mV s^–1^ scan rate from CV and 235.42 F g^–1^ at 0.5 A g^–1^ from GCD, significantly higher than those of pure mesoporous WO_3_ (142.18 F g^–1^ and 202.35 F g^–1^, respectively). Furthermore, the electrode retained 71% of its capacitance after 1000 charge–discharge cycles, demonstrating excellent cycling stability.

## Graphical abstract

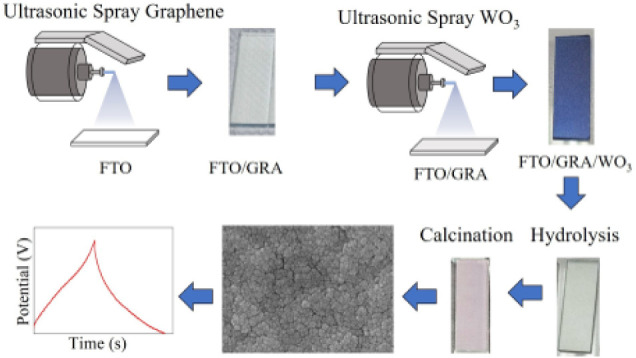

## 1 Introduction

With the continuous increase in global energy demand, fossil fuels are becoming insufficient to meet future requirements. Beyond their limited reserves, fossil fuels cause severe environmental degradation during extraction and refining processes, while their combustion releases significant amounts of greenhouse gases, such as carbon dioxide (CO_2_), thereby intensifying the greenhouse effect. Consequently, a global energy transition toward sustainable and renewable sources—such as solar, wind, and hydropower—has been widely promoted. These green energy sources are considered more desirable due to their environmental friendliness and renewability. However, their dependence on natural conditions results in intermittency and instability in power generation. Hence, to ensure a reliable and continuous energy supply, the development of efficient energy storage systems is indispensable.

Between 2012 and 2022, the number of publications related to supercapacitors in the energy storage field increased nearly 10-fold, indicating that supercapacitors have emerged as one of the most intensively studied research topics. Research in this area has expanded beyond electrode materials to include electrolytes and separators.^1^ Considerable efforts have focused on improving electrode performance through various strategies, particularly using carbon-based materials. Examples include the incorporation of transition metal oxides, sulfides, and their composites, as well as metal–organic frameworks (MOFs) and polyoxometalates (POMs). In addition, the development of two-dimensional materials such as MXenes and graphene has become another important research direction.^2^

Supercapacitors are generally categorized into two types based on their charge storage mechanisms: electric double-layer capacitors (EDLCs) and pseudocapacitors.^3^ EDLCs store energy through electrostatic charge accumulation at the electrode–electrolyte interface, typically using carbon-based materials such as activated carbon,^4^ carbon nanotubes,^5,6^ or graphene.^7,8^ In contrast, pseudocapacitors store energy through fast and reversible Faradaic redox reactions occurring on the electrode surface.^9^ Representative materials include transition metal oxides (TMOs) with multiple oxidation states, such as RuO_2_,^10^ MnO_2_,^11^ and WO_3_. Comparison with supercapacitor electrodes of different kinds is shown in Table 1.

**Table 1:** Comparison with Different Kinds of Supercapacitor Electrodes

Electrode Type	Mechanism	Key Materials
EDLC (Carbon)	Non-Faradaic Ion Adsorption (Double Layer)	Activated Carbon, Carbon Nanotubes (CNTs), Graphene
Pseudocapacitive (Metal Oxides)	Faradaic Redox Reactions	MnO_2_, RuO_2_, V_2_O_5_, NiO

In this study, tungsten oxide (WO_3_), a transition metal oxide, was employed as the primary active electrode material. WO_3_ possesses multiple oxidation states, high electrochemical stability, and a high theoretical specific capacitance, enabling favorable Faradaic charge storage characteristics that make it an excellent pseudocapacitive material. Although RuO_2_, one of the earliest pseudocapacitive materials, exhibits a wide potential window and high specific capacitance, its high cost, low crustal abundance, and scarcity significantly hinder its large-scale application.^12^ In comparison, WO_3_ offers several advantages, including low cost, low toxicity, and abundant natural reserves, making it a promising candidate for supercapacitor electrodes.^13^ Numerous studies have synthesized WO_3_-based electrodes through various methods;^14,15^ however, the experimentally obtained specific capacitance values remain far below theoretical expectations. This discrepancy primarily arises from WO_3_’s poor electrical conductivity, which results in high electron transport impedance and inferior rate capability.^16^

To overcome this limitation, this research proposed the incorporation of carbon materials with high conductivity and structural stability to form composite electrodes. Graphene, characterized by its low density, large specific surface area, excellent conductivity, and outstanding chemical stability,^17–21^ is an ideal candidate for this purpose. Many studies have reported graphene–metal oxide composites, such as graphene–MnO_2_ hybrids, which demonstrate enhanced electrochemical performance.^22,23^ For instance, Chen et al. deposited MnO_2_ nanoparticles on graphene oxide to significantly improve the electrochemical properties of the resulting nanocomposites.^24^ Subsequently, several studies have explored graphene–WO_3_ composites as electrode materials.^25,26^ Cai et al. prepared graphene oxide–WO_3_ composites via a precipitation method, achieving superior resistance and capacitance characteristics compared to pure WO_3_ electrodes.^13^

Furthermore, this work employed ultrasonic spray coating technology in combination with the sol–gel method for electrode film deposition. Conventional dip-coating techniques often suffer from uneven film distribution and high solvent consumption, whereas spin coating provides better film uniformity and lower solvent usage but faces challenges in scalability. Ultrasonic spray deposition, as an emerging technique, utilizes high-frequency nozzle vibration to generate fine aerosol droplets that are uniformly deposited on the substrate. This method combines the advantages of low solvent consumption (as in spin coating) and scalable production (as in dip coating).^27^ Additionally, process parameters can be easily adjusted via computer control, allowing precise regulation of film thickness through variation in spray passes or feed rates, thus enabling rapid optimization. Owing to these merits, ultrasonic spray deposition has been widely applied in photoelectrochemical fields,^28,29^ and it is expected to facilitate the fabrication of supercapacitor electrodes with superior electrochemical performance.

## 2 Experimental Section

### 2.1 Preparation of Graphene Solution

Graphene nanopowder (10 mg, UR-GRAPHENE, 8 nm flakes) was dispersed in 10 mL of anhydrous ethanol (≥99.5%) and subjected to ultrasonic agitation for 1 h to achieve a homogeneous suspension. The resulting graphene dispersion was then loaded into a syringe and installed on an ultrasonic spray deposition system for subsequent processing.

### 2.2 Preparation of WCl_6_-P123 Sol Solution

Since the precursor is highly sensitive to moisture, the preparation of the sol solution was conducted in a low-humidity environment. Poly­(ethylene oxide)-poly­(propylene oxide)-poly­(ethylene oxide) (P123, 1.5 g; Sigma-Aldrich) was weighed in a glovebox maintained at a relative humidity below 30%. The P123 was then dissolved in 10 mL of anhydrous ethanol (≥99.5%) under magnetic stirring for 30 min until complete dissolution was achieved. Subsequently, tungsten hexachloride (WCl_6_, 892.5 mg; ≥99.9%, Acros Organics) was added to the solution. After being continuously stirred for 12 h, the resulting sol was transferred to a syringe and mounted onto the ultrasonic spray system.

Upon the addition of WCl_6_ to the ethanol solution, the color gradually changed from being colorless and transparent to a distinct blue hue. This transformation is attributed to the redox interaction between WCl_6_ and ethanol, during which a portion of the W^6+^ ions is reduced to W^5+^. The resulting W^5+^-containing alkoxide species impart a characteristic blue coloration to the sol. The related reactions are shown below:^30,31^

1 $${\mathrm{WCl}}_{6}+2.5C_{2}H_{5}\mathrm{OH}\to {\mathrm{WCl}}_{3}{\left({\mathrm{OC}}_{2}H_{5}\right)}_{2}+0.5{\mathrm{CH}}_{3}\mathrm{CHO}+3\mathrm{HCl}$$

2 $$2{\mathrm{WCl}}_{3}{\left({\mathrm{OC}}_{2}H_{5}\right)}_{2}+2C_{2}H_{5}\mathrm{OH}\to W_{2}{\mathrm{Cl}}_{4}{\left({\mathrm{OC}}_{2}H_{5}\right)}_{6}+2\mathrm{HCl}$$

### 2.3 Fabrication of Graphene/Tungsten Oxide Thin Films

Prior to deposition, fluorine-doped tin oxide (FTO) glass substrates (TEC-15, Pilkington, 20 Ω sq^–1^) were sequentially cleaned with ethanol, acetone, deionized water, and ethanol under ultrasonic agitation for 3 min each. The cleaned substrates were then positioned in the spray zone beneath the nozzle.

The graphene dispersion, previously agitated for 1 h, was loaded into a syringe connected to a syringe pump (YSC, SP series), which delivered the sol to the ultrasonic nozzle at a constant flow rate of 0.25 mL min^–1^. The ultrasonic spray system consisted of a benchtop ultrasonic spray coater (Dispensing Technology, DT-300), an ultrasonic nozzle (WHIRL BEST, 120 kHz), and an ultrasonic spray controller (WHIRL BEST). A gas delivery system equipped with a flow controller (Cylinder World) supplied nitrogen gas at a rate of 6.9 slm to transport the atomized sol onto the FTO substrate. The nozzle operated at a vibration frequency of 120 kHz with a power input of 4 W. The overall fabrication procedure is demonstrated in Scheme 1.

**Scheme 1:**
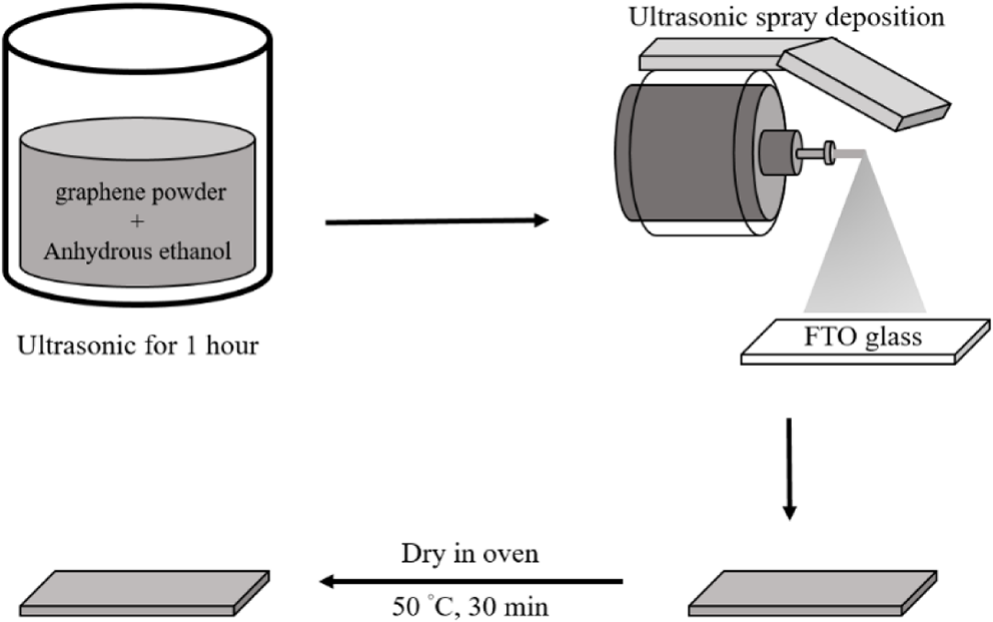
Schematic Diagram of Graphene Film Preparation

The FTO/GRA substrate was subsequently placed in the spraying stage. The sol, which had been magnetically stirred for 12 h, was transferred to a syringe and delivered to the ultrasonic nozzle at a constant flow rate of 0.25 mL min^–1^ using the syringe pump. Following deposition, an iridescent blue film was observed on the surface of the FTO glass. The as-sprayed sample was then transferred to a sealed container saturated with water vapor to induce hydrolysis.

Given the high hydrolysis sensitivity of transition metal alkoxides, exposure to a water vapor-saturated environment effectively promotes the hydrolysis process. During this stage, the sol undergoes hydrolysis in the presence of moisture and oxygen, leading to the formation of tungsten hydroxide (WO­(OH)­6_–*x*_). This process is typically accompanied by a gradual color change of the film. After 12 h of hydrolysis, the film’s appearance transitioned from an iridescent blue to a transparent state, indicating the completion of hydrolysis. The overall reaction can be represented as follows:^32^

3 $$M-\mathrm{OR}+H_{2}O\to M-OH+\mathrm{ROH},,M=\mathrm{WC}l_{3}{\left(OC_{2}H_{5}\right)}_{2}\;\mathrm{or}\;W_{2}Cl_{4}{\left(OC_{2}H_{5}\right)}_{6}$$

The final step involved a calcination process. After 12 h of hydrolysis, the sample was placed on a heated plate and annealed in air at 350 °C for 1 h. This thermal treatment was served to remove the P123 template, complete the oxidation, and crystallize the WO_3_. The overall fabrication procedure is illustrated in Scheme 2.

**Scheme 2:**
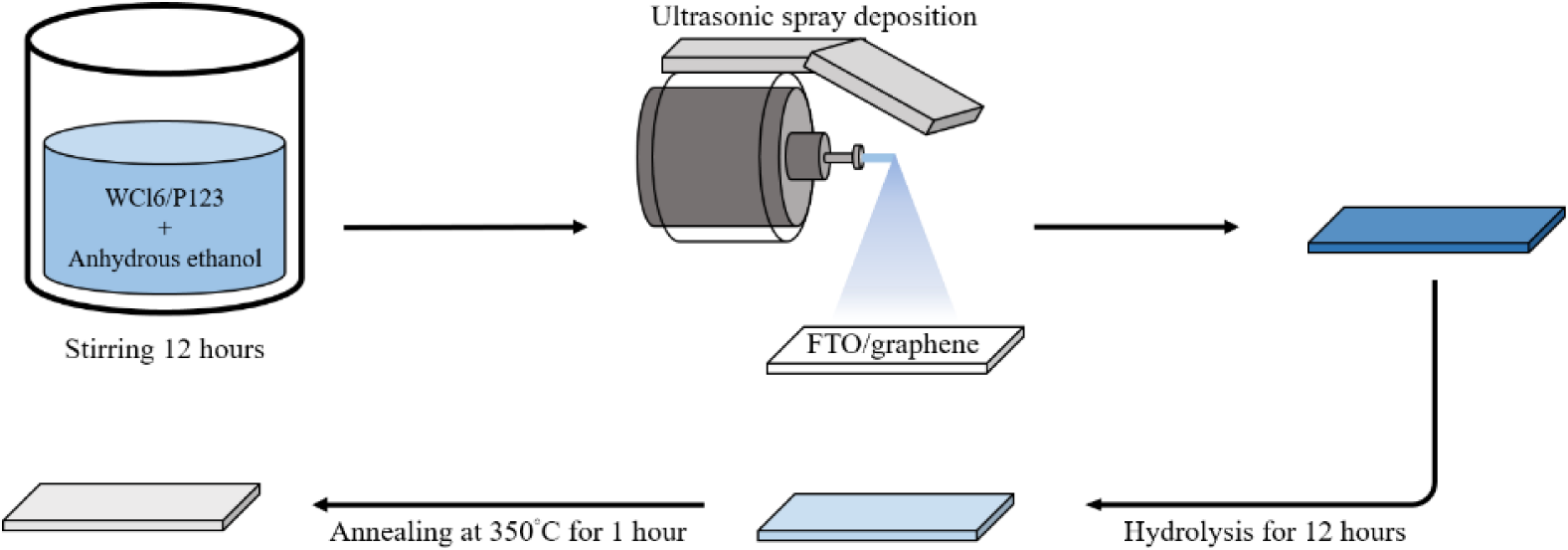
Schematic Diagram of the WO_3_ Film Preparation

### 2.4 Characterization of Deposited Films

The crystallinity of the films was analyzed by grazing-incidence X-ray diffraction (GIXRD) using Cu Kα radiation (λ = 1.5406 Å). Diffraction patterns were collected over a 2θ range of 10–65° with a step size of 0.02° and a scan rate of 2° min^–1^. Raman spectroscopy was conducted using a 588 nm excitation laser to identify molecular vibrations and confirm the phase composition of the films. The surface morphology and microstructural characteristics were examined using a scanning electron microscope (JSM-5600), and the elemental composition and spatial distribution were further investigated by energy-dispersive X-ray spectroscopy (EDX). Nitrogen physisorption was carried out by utilizing a Micromeritics ASAP 2020 instrument after the samples were degassed at 250 °C under vacuum for 4 h.

Electrochemical measurements were carried out by using a Biologic SP-150 potentiostat in a three-electrode configuration. The experimental setup for electrochemical measurement is shown in Scheme 3. The as-prepared film served as the working electrode, immersed in a 1 M lithium perchlorate (LiClO_4_) solution prepared in propylene carbonate (PC). A saturated Ag/AgCl electrode and a platinum wire were used as the reference and counter electrodes, respectively. All electrochemical tests were performed at room temperature under ambient atmospheric conditions.

**Scheme 3:**
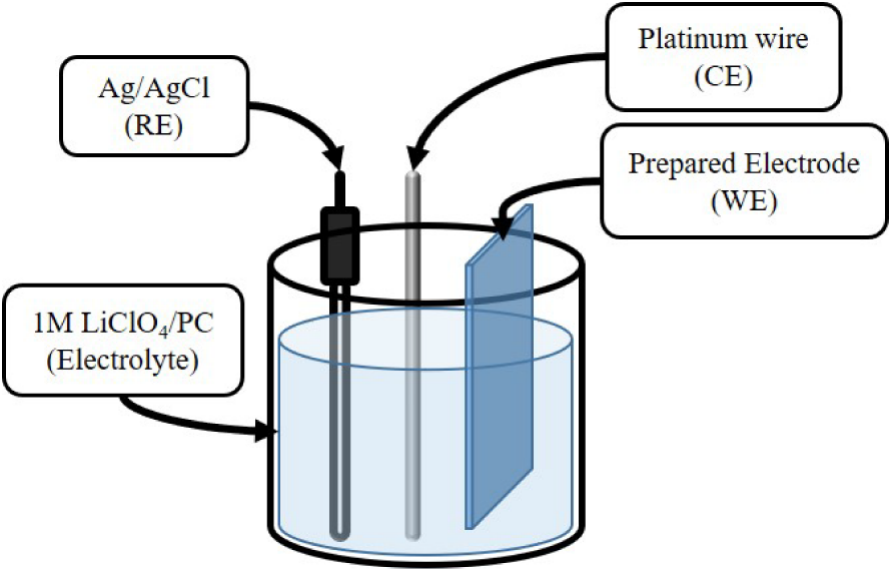
Experimental Setup of Electrochemical Measurement

## 3 Results and Discussion

### 3.1 Surface Morphology of Graphene/Mesoporous Tungsten Oxide Thin Films

Figure 1 presents the surface morphologies of the graphene sheets distributed on the FTO glass substrate. As shown in Figure 1a, the film deposited by ultrasonic spray coating retains a distinct sheet-like morphology, indicating that the graphene structure remains intact after deposition. In Figure 1b, the surface morphology exhibits a clearly smooth texture over a large area, which is attributed to the uniform deposition of tungsten oxide nanocrystals on the graphene sheets. The nanocrystals are homogeneously dispersed across the graphene surface, forming a continuous, uniform, and well-integrated composite layer.

**Figure 1:**
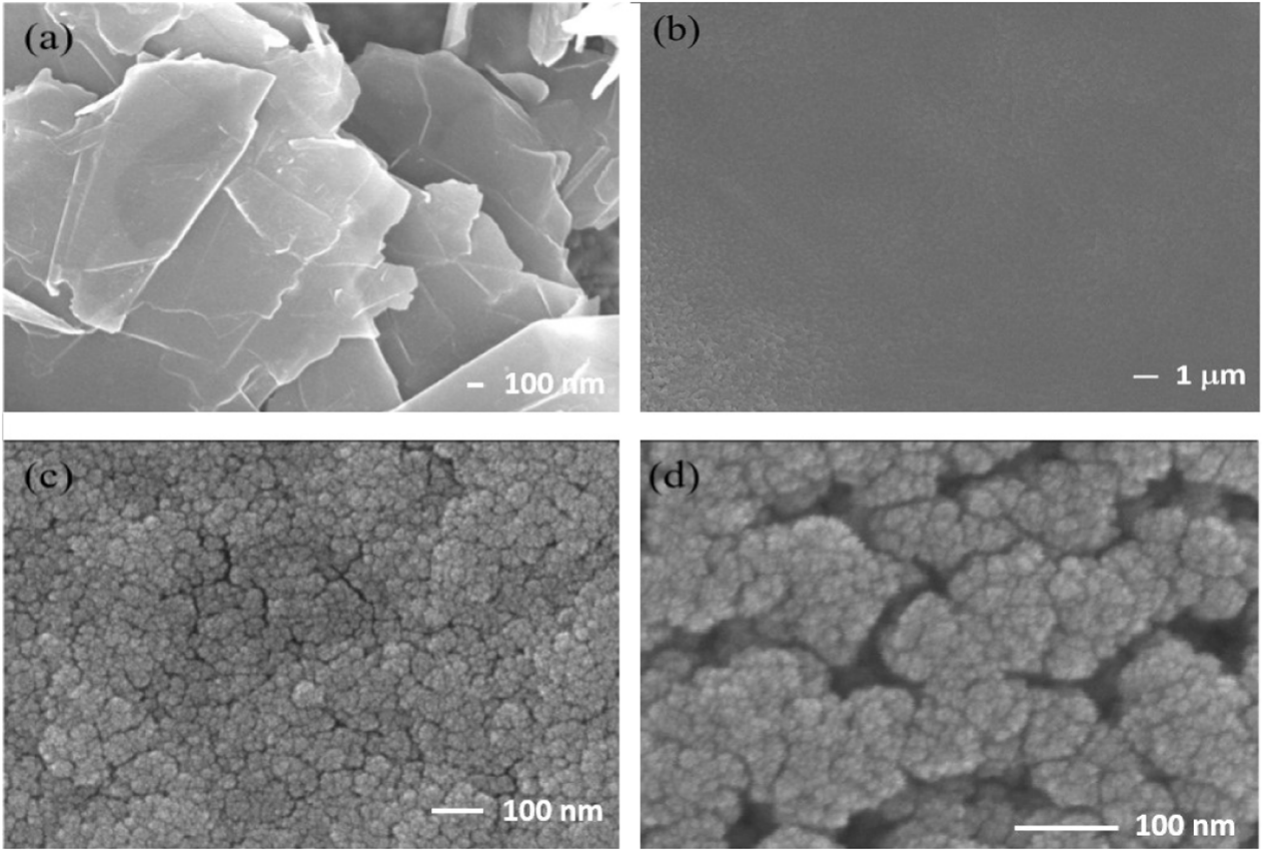
Surface morphology of (a) FTO/GRA, (b–d) FTO/GRA/WO_3_.

Figure 1c,d displays high-magnification SEM images (100,000× and 200,000×, respectively) of the tungsten oxide nanocrystals grown on the graphene sheets. The images reveal that the interconnected nanocrystals stack together to form a porous tungsten oxide thin film. The WO_3_ coating on the composite electrode surface exhibits a highly porous morphology with uniformly distributed mesopores, most of which are smaller than 50 nm in diameter.

The formation of this mesoporous structure is primarily attributed to the use of the triblock copolymer Pluronic P123 as a soft template during synthesis. The P123 molecular chains contain both hydrophilic and hydrophobic segments, which undergo spontaneous microphase separation in the solvent due to their mutual incompatibility and covalent bonding constraints. During gelation of the tungsten oxide precursor, the P123 micelles interact with the precursor species, directing the self-assembly into an ordered mesostructure. Upon calcination, the P123 template is thermally decomposed and removed, resulting in a continuous and open mesoporous WO_3_ network. This templated synthesis strategy effectively increases the specific surface area and porosity of the WO_3_ thin film, thereby enhancing its suitability for electrochemical energy storage applications.

As shown in Figure 2, the cross-sectional morphology of the composite electrode film on the FTO conductive glass substrate was examined by using field-emission scanning electron microscopy (FE-SEM). The image reveals a well-defined multilayered structure consisting of 16 sprayed graphene layers and 4 mesoporous tungsten oxide layers, with a total film thickness of approximately 0.5 μm. This structural configuration was determined to be the optimized condition for achieving superior electrochemical performance in this study.

**Figure 2:**
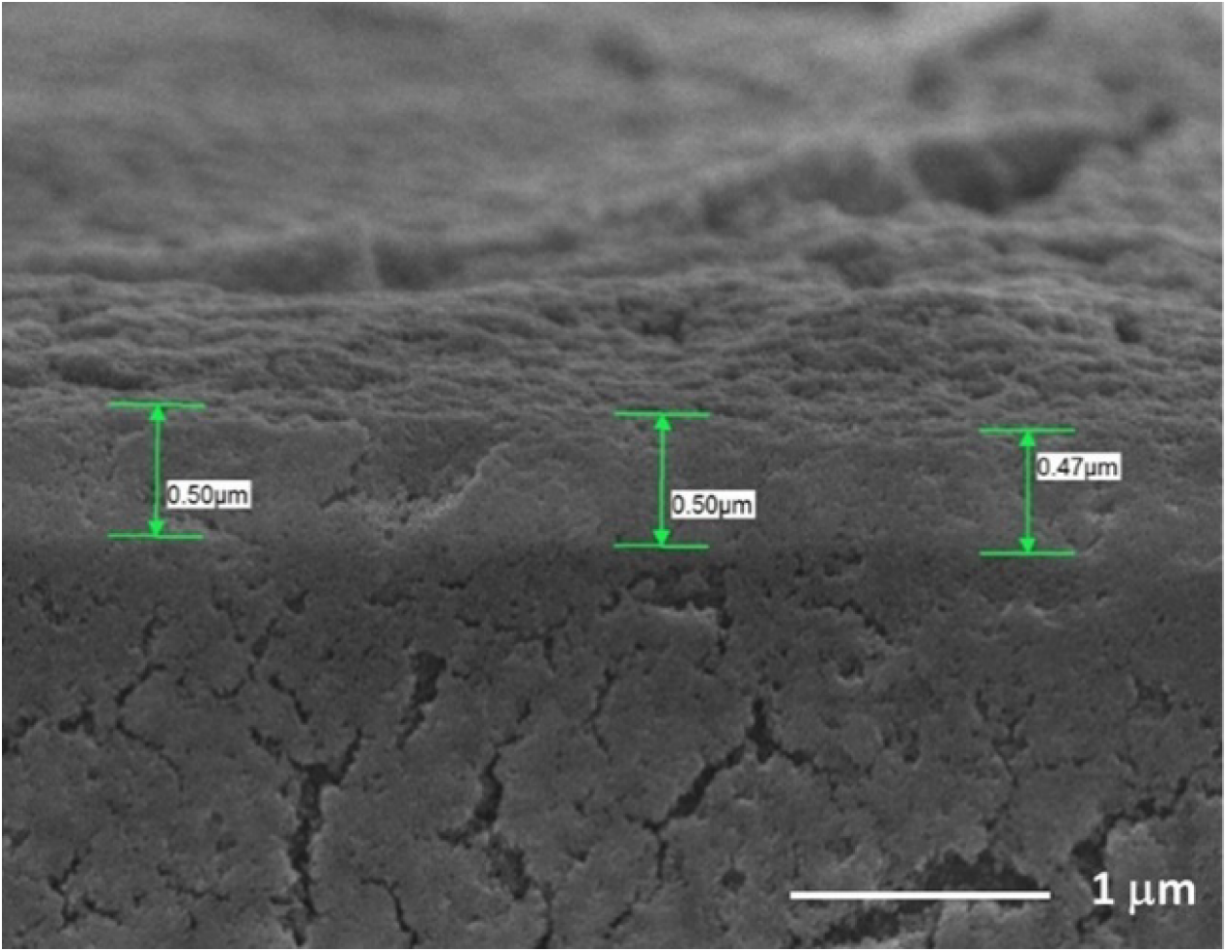
SEM cross-sectional view of the FTO/GRA/WO_3_.

### 3.2 Structural Characterization of Mesoporous Tungsten Oxide Thin Films

Figure 3a demonstrates the nitrogen physisorption isotherms, and Figure 3b displays the pore size distributions and specific surface area of GRA/WO_3_. The shapes of the isotherm plots of the films in this research match with type IV of the BET classification, indicating the characteristics of mesoporous materials.^33–35^ The pore size of the composite is around 4–10 nm with a specific surface area of 116 m^2^/g.

**Figure 3:**
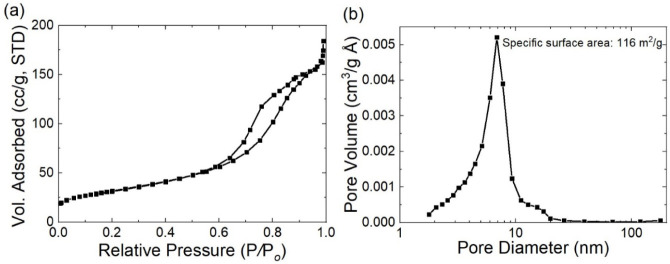
(a) Nitrogen physisorption isotherms and (b) pore size distributions and specific surface area of GRA/WO_3_.

To further examine the elemental composition and spatial distribution within the composite electrode, field-emission scanning electron microscopy (FE-SEM) coupled with energy-dispersive X-ray spectroscopy (EDX) and elemental mapping (EDX mapping) was performed on the sample deposited on the FTO substrate. The EDX spectra confirmed the presence of tungsten (W), oxygen (O), and carbon (C) originating from tungsten oxide and graphene as well as tin (Sn) and fluorine (F) from the underlying FTO substrate. These results verify the successful integration of both WO_3_ and graphene within the composite film. Moreover, the EDX elemental mapping images reveal a uniform spatial distribution of the constituent elements across the electrode surface, indicating homogeneous deposition and strong interfacial contact between the graphene and tungsten oxide layers.

As shown in Figure 4 and summarized in Table 2, the atomic ratio of tungsten to oxygen on the electrode surface remains approximately 1:3, regardless of whether graphene nanosheets were incorporated. This stoichiometric ratio confirms that the material deposited on the FTO glass substrate corresponds to tungsten trioxide (WO_3_). In the composite sample containing graphene, carbon signals were also detected, further validating the successful incorporation of graphene nanosheets onto the FTO substrate. Figure 5 shows the elemental mapping results of the FTO/GRA/WO_3_ composite electrode obtained by using field-emission scanning electron microscopy (FE-SEM) coupled with energy-dispersive X-ray spectroscopy (EDX). The mapping images reveal that all detected elements are uniformly distributed across the electrode surface, indicating that the film fabricated by ultrasonic spray deposition exhibits excellent compositional uniformity and deposition controllability.

**Figure 4:**
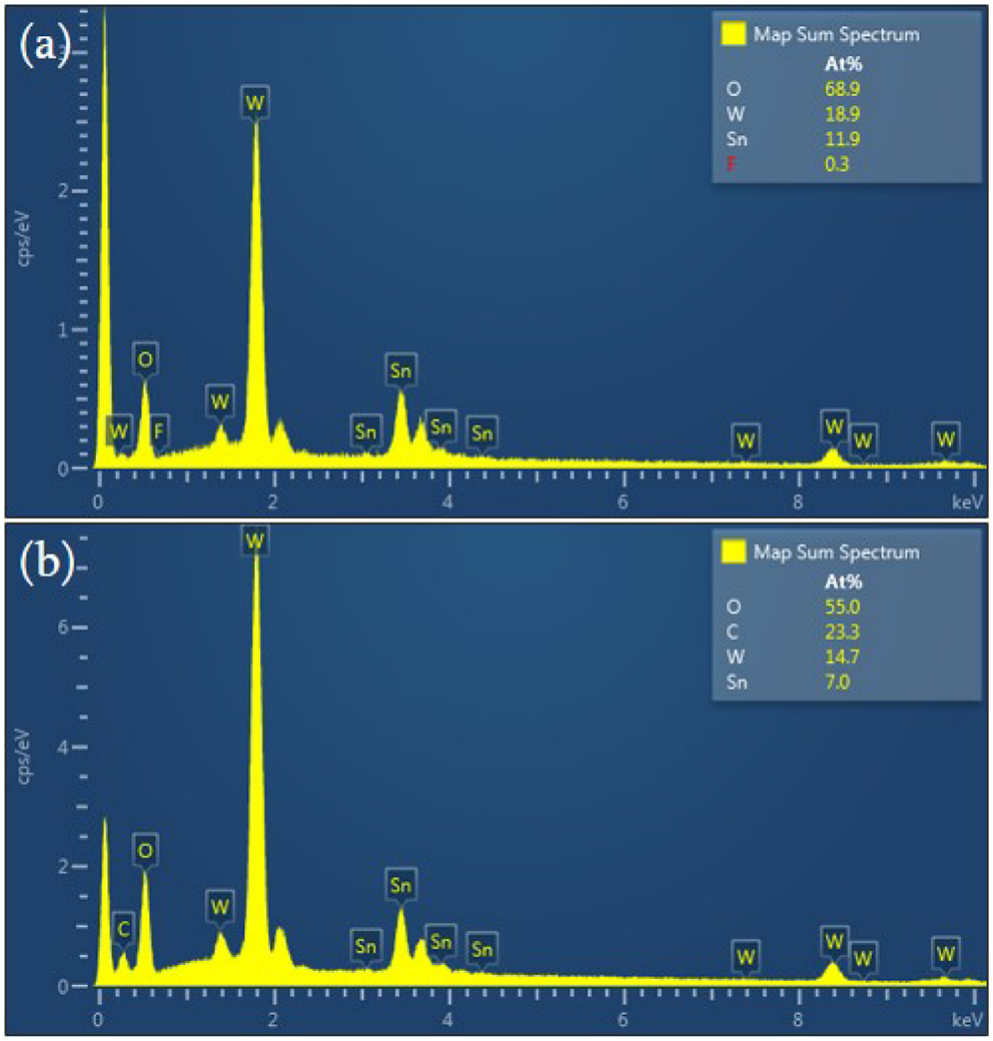
EDX spectra of (a) FTO/WO_3_ and (b) FTO/GRA/WO_3_.

**Table 2:** Elemental Compositions of FTO/WO_3_ and FTO/GRA/WO_3_ Electrodes

Sample	W (at. %)	O (at. %)	C (at. %)	Sn (at. %)	F (at. %)
FTO/WO_3_	18.9	68.9		11.9	0.3
FTO/GRA/WO_3_	14.7	55	23.3	7	

**Figure 5:**
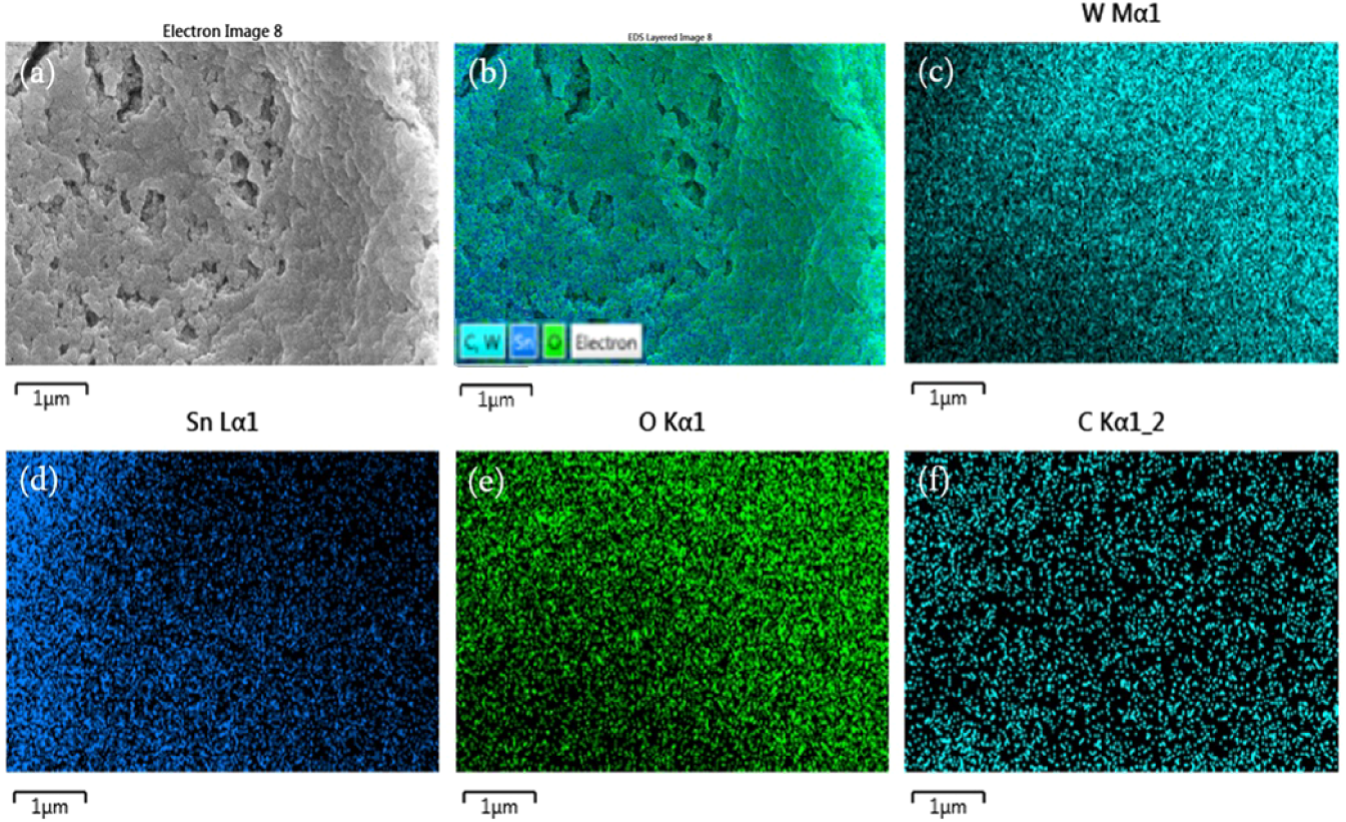
EDX elemental mapping of FTO/GRA/WO_3_ electrode. (a) Electron image, (b) EDX image, (c) tungsten element image, (d) tin element image, (e) oxygen element image, and (f) carbon element image.

The GRA/WO_3_ films prepared via ultrasonic spray deposition (USD) combined with the template-assisted sol–gel method were characterized by using X-ray diffraction (XRD, Figure 6) and Raman spectroscopy (Figure 7), both confirming the formation of a monoclinic phase. To minimize signal interference from the FTO conductive glass substrate, grazing-incidence X-ray diffraction (GIXRD) was employed for structural analysis.

**Figure 6:**
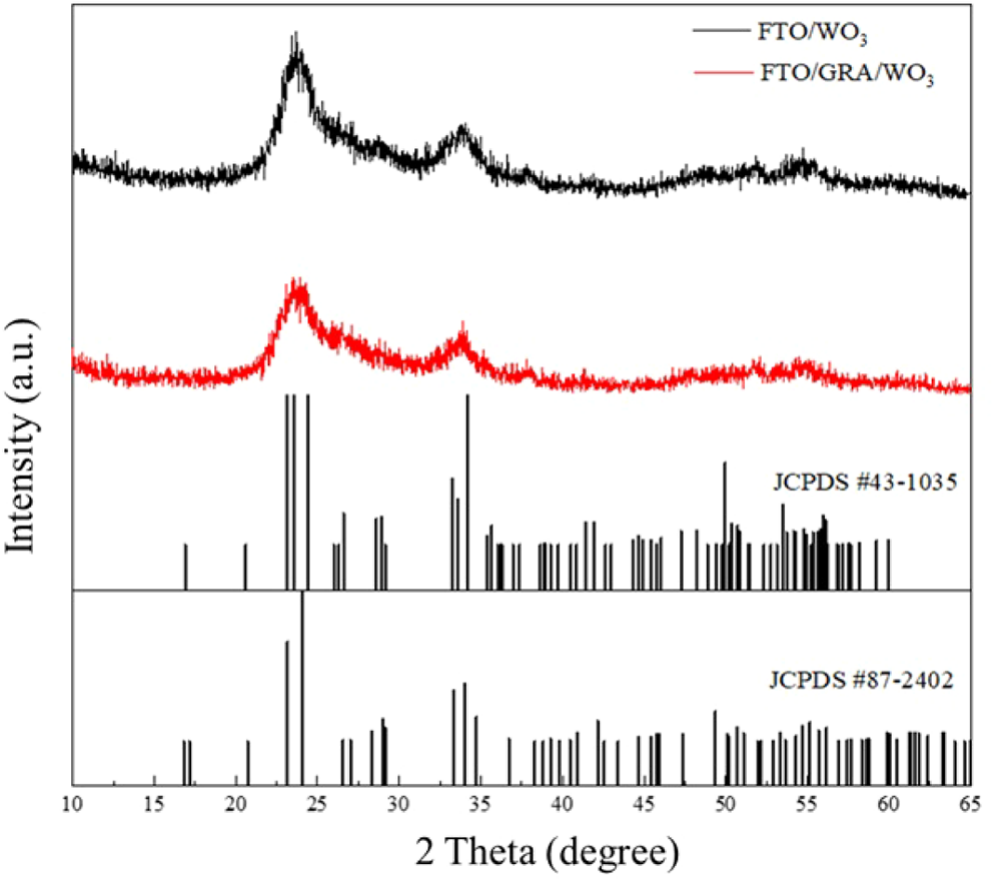
XRD patterns of FTO/WO_3_ and FTO/GRA/WO_3_.

**Figure 7:**
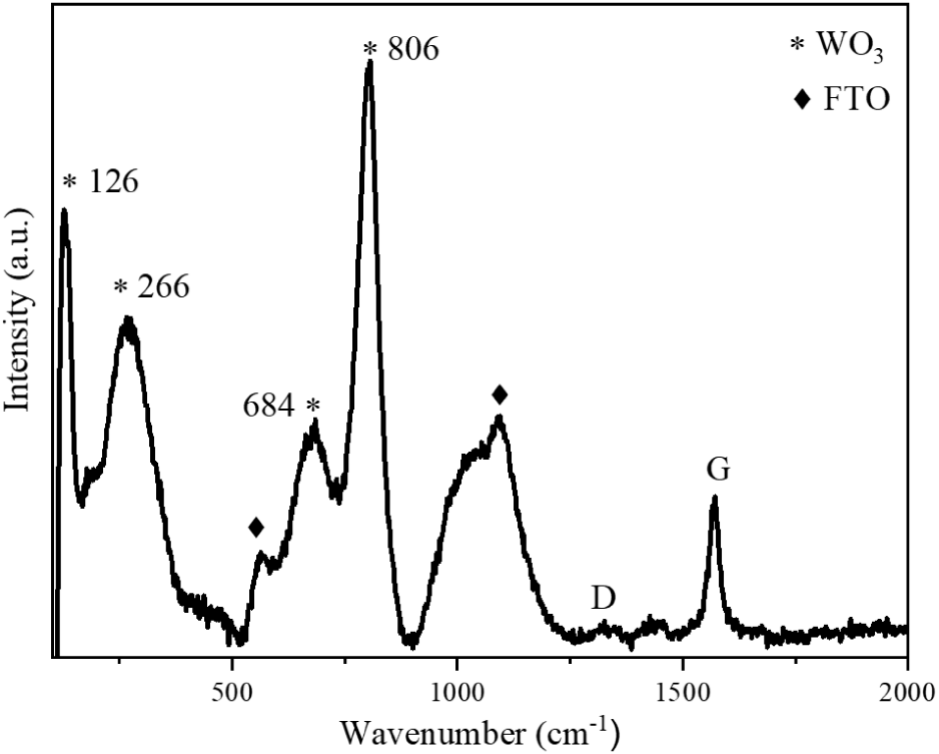
Raman spectra of FTO/GRA/WO_3_.

The XRD patterns exhibit diffraction peaks corresponding to the γ-phase (JCPDS No. 43-1035) and ε-phase (JCPDS No. 87-2402) of monoclinic WO_3_, in good agreement with previously reported data.^36^ The broad and relatively weak diffraction peaks are attributed to the mesoporous characteristics of the films, which reduce the long-range crystallographic order. Notably, the introduction of a graphene interlayer between the FTO and WO_3_ results in characteristic graphene reflections appearing in the 2θ range of 20°–30°. However, due to overlapping signals between graphene and WO_3_, the graphene peak intensity is significantly diminished—consistent with the observations reported by Firat .^37^ In our previous study,^38^ the monoclinic phase of WO_3_ demonstrated better electrochemical performance which agrees with other literature.^39^ The monoclinic phase WO_3_ was chosen in this research as the electrode material for supercapacitor evaluation.

The Raman spectra (Figure 7) are consistent with the XRD results, further confirming the monoclinic phase of WO_3_. The peak observed at 126 cm^–1^ is attributed to lattice vibrations, while the characteristic peaks of the γ-monoclinic WO_3_ phase appear at approximately 266, 684, and 806 cm^–1^. Additional peaks located near 563 and 1091 cm^–1^ originate from the FTO glass substrate. Prominent peaks at 1335 and 1570 cm^–1^ correspond to the D and G bands of graphene, respectively, confirming the successful incorporation of graphene within the composite electrode.

The calculated intensity ratio of the D to G bands (*I*_D_/*I*_G_) is approximately 0.14, indicating a low defect density within the graphene structure.^40^ This result is consistent with the use of commercially available graphene nanopowder and the absence of additional chemical modification steps during electrode fabrication, both of which contribute to the preservation of the graphene’s structural integrity.

### 3.3 Electrochemical Measurements of Mesoporous Tungsten Oxide Thin Films

To evaluate the capacitive performance of the GRA/WO_3_ films fabricated via the two-step ultrasonic spray deposition process, electrochemical measurements were performed in a 1 M LiClO_4_/propylene carbonate (PC) electrolyte. A platinum wire and a silver/silver chloride (Ag/AgCl) electrode were employed as the counter and reference electrodes, respectively. Cyclic voltammetry (CV) and galvanostatic charge–discharge (GCD) tests were carried out at room temperature within a potential window ranging from 0 to −1.5 V.

The mass of the active material (WO_3_ or GRA/WO_3_) deposited on the FTO substrate was determined by measuring the weight difference of the FTO glass before and after deposition by using an analytical balance. The specific capacitance (*C*) values obtained from CV and GCD measurements were calculated using eqs 4 and 5, respectively.

4 $$C=\frac{\int I\left(V\right)\;dV}{2mv\Delta V}$$

5 $$C=\frac{It}{m\Delta V}$$

Figure 8 presents the electrochemical performance of mesoporous tungsten oxide films prepared with 3, 4, 5, and 6 sprayed layers. The specific capacitance values of the electrodes were calculated from the cyclic voltammetry (CV) curves using the corresponding equation, where *v* represents the scan rate (10 mV s^–1^), *m* denotes the mass of the active material (g), and Δ*V* is the potential window.

**Figure 8:**
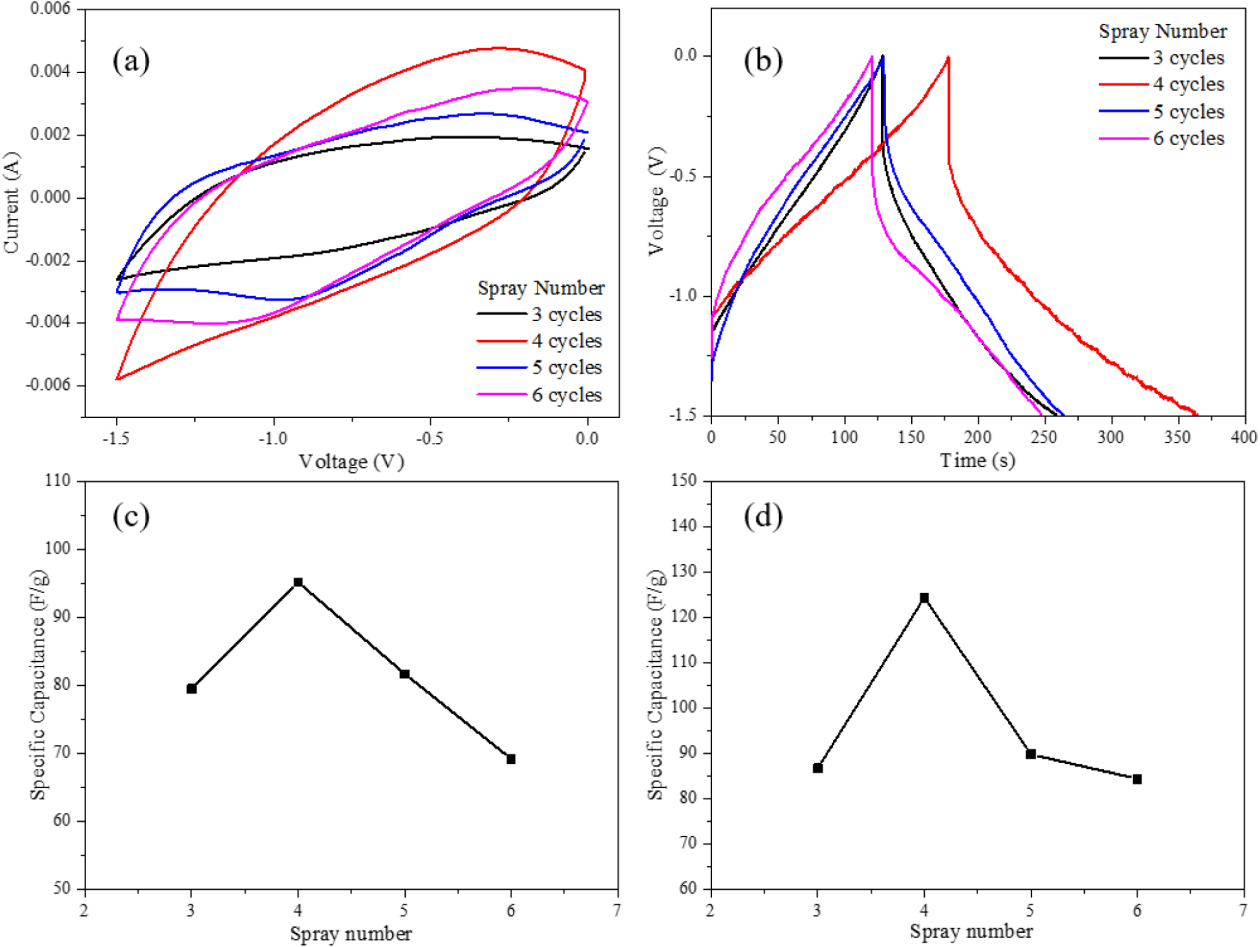
(a) CV curve of FTO/WO_3_ with different spray coats at a constant scan rate of 10 mV/s, (b) GCD curves of FTO/WO_3_ with different spray coats at a constant current density of 1 A g^–1^ and the relationship between specific capacitance and spray coats at (c) CV and (d) GCD.

The calculated specific capacitances for the electrodes with 3, 4, 5, and 6 sprayed layers were 79.53, 95.23, 81.72, and 69.21 F g^–1^, respectively. As the film thickness increased from 3 to 4 layers, the specific capacitance improved due to the increased amount of electroactive material and enhanced ion accessibility. However, further increasing the film thickness beyond 4 layers resulted in a reduction in specific capacitance, likely caused by the longer ion diffusion paths and higher internal resistance associated with excessive film thickness.

Based on the galvanostatic charge–discharge (GCD) analysis, the specific capacitance was calculated according to the corresponding equation with the current density (*I*) fixed at 1 A g^–1^. The resulting specific capacitances for the electrodes with 3, 4, 5, and 6 sprayed layers were 86.77, 124.43, 89.86, and 84.43 F g^–1^, respectively. The pseudocapacitive behavior of WO_3_ originates from the surface ion adsorption/desorption processes coupled with the reversible redox transition between W^6+^ and W^5+^ oxidation states. The electrochemical reaction can be expressed as follows:

6 $$WO_{3}+xM^{+}+xe^{-}\to M_{x}WO_{3}$$

The four-layer sprayed WO_3_ film exhibited a higher specific capacitance than the three- and five-layer samples. The decline observed in the five-layer film is attributed to excessive film thickness, which impedes ion adsorption and desorption processes—an effect consistent with previous reports.^36^

To further enhance the electrochemical performance of the mesoporous WO_3_ film electrode, graphene nanopowder was introduced as a conductive interlayer between the FTO substrate and the mesoporous WO_3_ layer. Owing to its outstanding electrical conductivity and high specific surface area, graphene effectively facilitates electron transport and promotes interfacial charge transfer, thereby improving both specific capacitance and rate capability.

In this study, different numbers of graphene layers (12, 16, 20, and 24) were deposited between the FTO substrate and the WO_3_ film to investigate the influence of interlayer thickness on the electrode’s capacitive behavior. Figure 9 presents the electrochemical performance of the composite electrodes after incorporation of the graphene interlayer.

**Figure 9:**
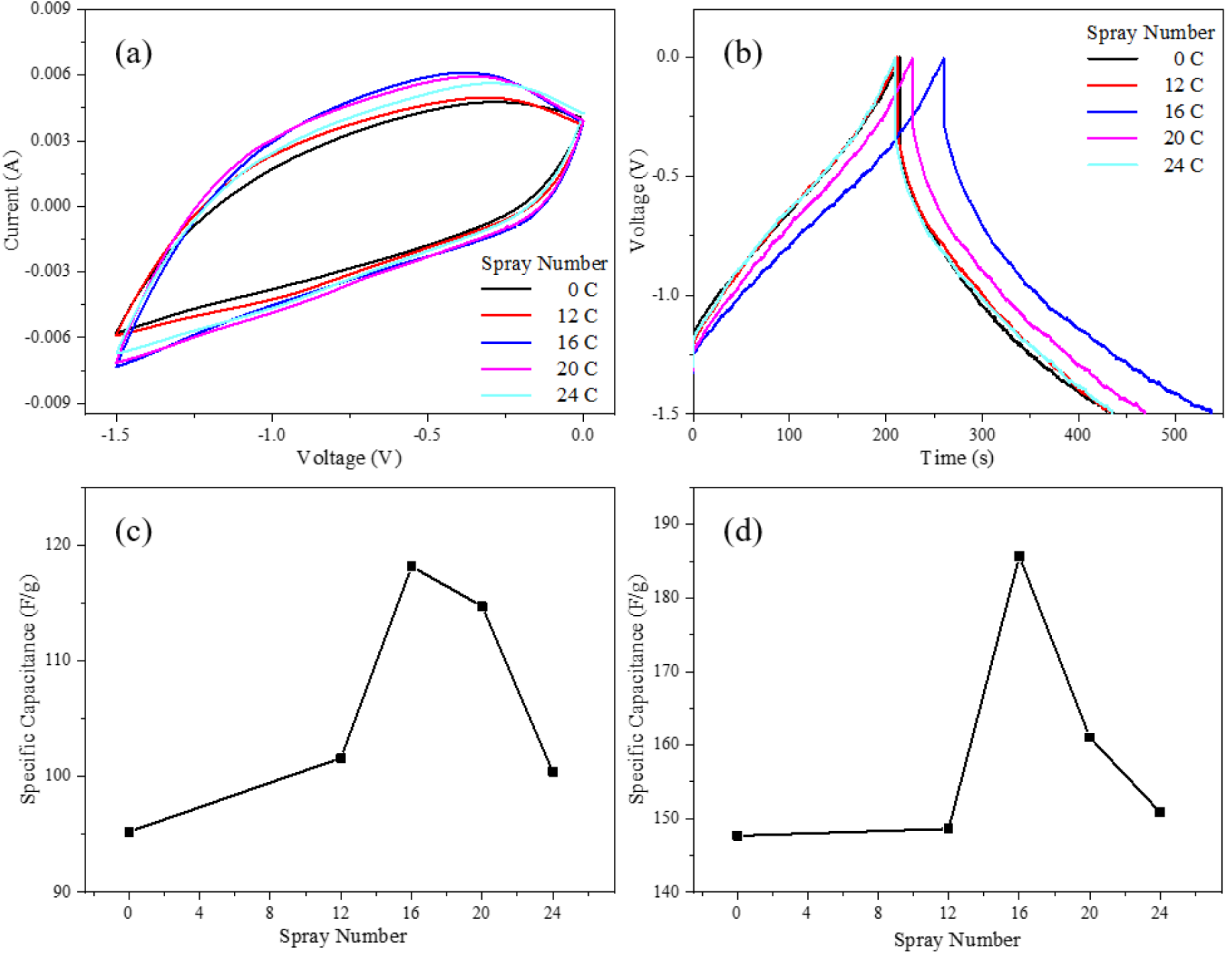
(a) CV curve at a constant scan rate of 10 mV/s, (b) GCD curves at a constant current density of 1 A g^–1^ of FTO/GRA/WO_3_ with different graphene spray coats, and the relationship between specific capacitance and spray coats at (c) CV and (d) GCD.

Based on the same calculation formula, the specific capacitances of electrodes composed of 4 layers of mesoporous WO_3_ combined with 0, 12, 16, 20, and 24 sprayed graphene layers at a fixed scan rate of 10 mV s^–1^ were 95.23, 101.61, 118.19, 114.73, and 100.42 F g^–1^, respectively.

To further validate the cyclic voltammetry (CV) results, galvanostatic charge–discharge (GCD) measurements were performed on the same set of electrodes. The results consistently showed that the sample incorporating 16 graphene layers exhibited the longest charge–discharge duration and the highest specific capacitance. The specific capacitances obtained from GCD for the electrodes with 0 (pure WO_3_), 12, 16, 20, and 24 graphene layers were 147.67, 148.54, 185.69, 161.06, and 150.85 F g^–1^, respectively. These findings align with the CV results, confirming that the electrode with 16 graphene layers achieved optimal electrochemical performance. The relationship between layers and capacitance of WO_3_ and GRA with 4 layers WO_3_ were summarized in Table 3.

**Table 3:** Relationship between Layers and Capacitance of WO_3_ and GRA with 4 Layers WO_3_

Materials	WO_3_		GRA + 4 layers WO_3_		
Method	CV	GCD	Method	CV	GCD
WO_3_ layers	Capacitance (F g^–1^)		GRA layers	Capacitance (F g^–1^)	
3	79.53	86.77	0	95.23	147.67
4	95.23	124.43	12	101.61	148.54
5	81.72	89.86	16	118.19	185.69
6	69.21	84.43	20	114.73	161.06
-	-	-	24	100.42	150.85

The comparison of electrochemical behavior of the optimized electrode (16 graphene layers and 4 mesoporous WO_3_ layers) with that of the pure mesoporous WO_3_ electrode is shown in Figure 10. Measurements were conducted within a potential window of −1.5 to 0.0 V at scan rates of 5, 10, 20, 50, and 100 mV s^–1^. The specific capacitance values of the pure WO_3_ electrode were 142.18, 95.23, 53.16, 16.04, and 5.63 F g^–1^, respectively, while those of the graphene-modified electrode were 161.68, 118.19, 69.20, 29.97, and 10.67 F g^–1^, respectively. It is evident that the incorporation of graphene significantly enhanced the specific capacitance across all scan rates, yielding improvements ranging from 13% to 90%, particularly at higher scan rates where charge transfer kinetics are more critical. From the shape of the CV and GCD plots, GRA/WO_3_ films showed more surface redox reaction (pseudocapacitance) contributions, rather than EDLCs.^9^

**Figure 10:**
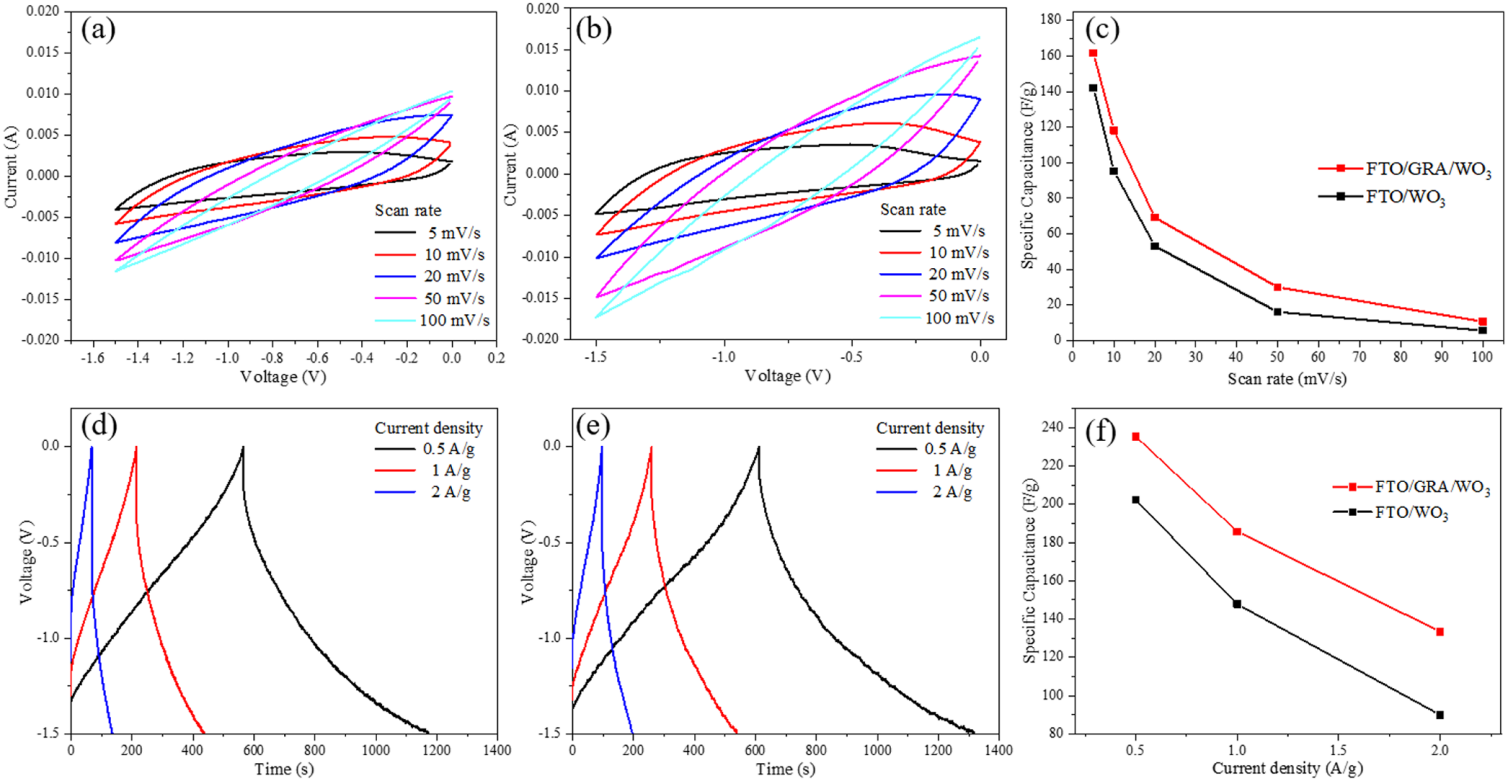
CV curve of (a) FTO/WO_3_ and (b) FTO/GRA/WO_3_ with different scan rates. (c) The plot of CV specific capacitance varied with current densities. GCD curve of (d) FTO/WO_3_ and (e) FTO/GRA/WO_3_ with different current densities. (f) The plot of GCD specific capacitance varied with current densities.

Further galvanostatic charge–discharge (GCD) measurements were conducted within the same potential window at current densities of 0.5, 1, and 2 A g^–1^ to evaluate the actual energy storage performance of the electrodes. The specific capacitances of the pure WO_3_ electrode at these current densities were 202.35, 147.67, and 89.88 F g^–1^, respectively, whereas those of the graphene-modified electrode were 235.42, 185.69, and 133.36 F g^–1^, respectively. In all cases, the graphene-modified electrode exhibited a higher specific capacitance, demonstrating enhanced electrochemical performance and improved charge transfer kinetics across various current densities.

To benchmark the capacitance performance against previously reported WO_3_-based supercapacitor electrodes, relevant literature data are summarized in Table 4. Compared with electrodes fabricated by electrodeposition (Firat^37^), chemical bath deposition (Korkmaz^41^), electrostatic self-assembly (Wong^43^), and simple deposition (Cai^13^), the electrode developed in this study—prepared via a sol–gel approach combined with ultrasonic spray deposition—exhibited superior specific capacitance. Differences among these studies can also be attributed, in part, to variations in the electrolytes and testing conditions employed. The main reason why the performance of this study is better than that of the literature in Table 4 is that the WO_3_ deposited using USD and the sol–gel method is mainly composed of nanocrystals and thus has a higher specific surface area.

**Table 4:** Comparison of Capacitance Values from This Work and Previous Studies

Material	Method	Potential Range (V)	Electrolyte	Scan rate (mV/s)	CV specific capacitance (F g^–1^)	Current density (A g^–1^)	GCD Specific Capacitance (F g^–1^)	Refs.
GRA/WO_3_	Sol–gel and USD	–1.5–0.0	1 M LiClO_4_/PC	5	161.68	0.5	235.42	This work
rGO-WO_3_	Electrodeposition	–0.7–0.2	1 M H_2_SO_4_	5	49.3	1	58.3	^37^
GNS-W	Facile precipitation	0.0–1.0	1 M H_2_SO_4_			0.1	143.6	^13^
GO/WO_3_.	CBD	–0.2–1.2		25	158.5			^41^
WO_3_·H_2_O/rGO	Hydrothermal	–0.4–0.1	1 M H_2_SO_4_			1	244	^42^
rGO/WO_3_	Electrostatic assembly	–1.0–1.0	1 M Na_2_SO_4_			0.7	85.7	^43^

Most previous studies employed highly concentrated strong acids, such as sulfuric acid (H_2_SO_4_) or hydrochloric acid (HCl), as electrolytes. Although these electrolytes provide abundant hydrogen ions and favorable redox conditions for WO_3_-based electrodes, their strong corrosiveness poses safety risks to both the equipment and operators. Moreover, they are difficult to recycle and environmentally unfriendly, which conflicts with the current pursuit of green and sustainable energy technologies.

In contrast, the present study utilized lithium perchlorate (LiClO_4_) as the electrolyte. While lithium ions are larger and exhibit slower diffusion rates compared to protons in strong acid electrolytes, LiClO_4_ offers superior chemical stability, safer operation, and greater environmental compatibility, making it highly suitable for sustainable energy storage applications. Notably, Ma et al.^42^ fabricated WO_3_·H_2_O/rGO electrodes via a hydrothermal method using strong acid electrolytes and achieved performance comparable to that of the present FTO/GRA/WO_3_ electrodes. This finding highlights the strong potential of the sol–gel-assisted ultrasonic spray deposition method employed in this work for producing high-performance, environmentally friendly supercapacitor electrodes.

To further elucidate the relationship between film thickness and internal resistance in both mesoporous WO_3_ and graphene-modified composite electrodes, electrochemical impedance spectroscopy (EIS) measurements were conducted, as shown in Figure 11. The tests were performed using a 1 M LiClO_4_/PC electrolyte at room temperature over a frequency range of 40 kHz to 100 mHz with a Biologic SP-150 potentiostat. Both samples exhibited similar series resistance values (∼15 Ω) under identical electrolyte conditions. However, in the magnified high-frequency region, the semicircular diameter of the Nyquist plot for the FTO/WO_3_ electrode (red curve) was significantly larger than that of the FTO/GRA/WO_3_ electrode (black curve). The fitted charge transfer resistances (*R*_ct_) were 45.01 and 22.46 Ω, respectively—approximately a 2-fold reduction after introducing the graphene layer. This demonstrates that graphene effectively enhances electron transport and facilitates faster charge transfer within the composite electrode.

**Figure 11:**
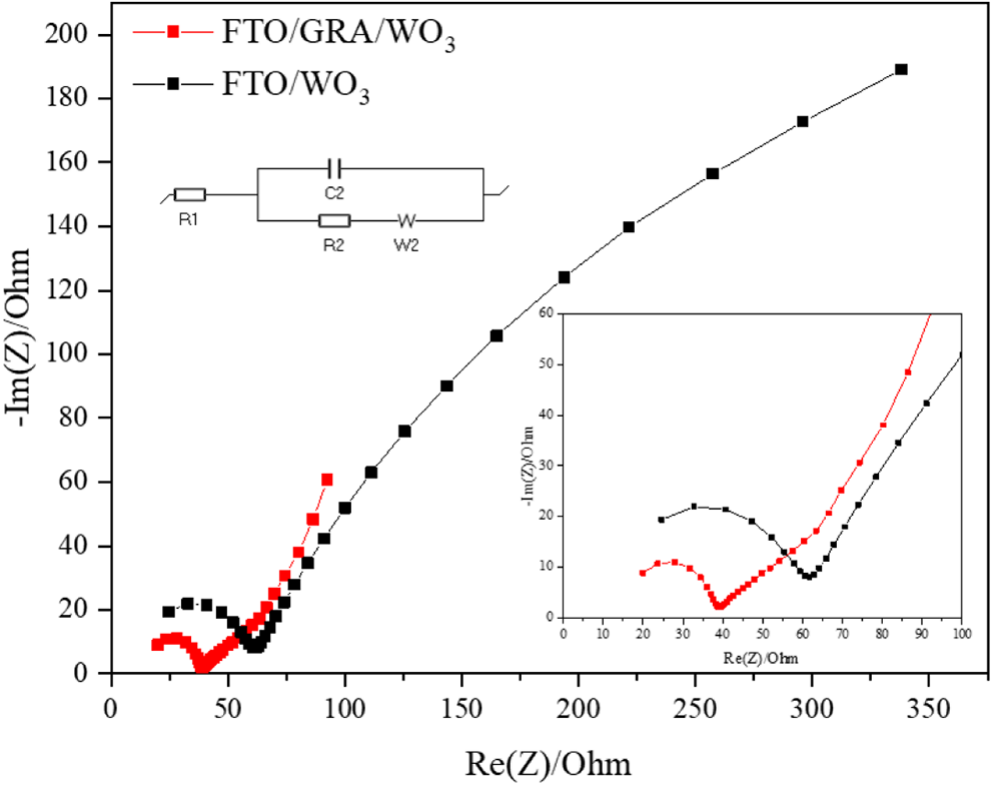
Nyquist plots of the FTO/WO_3_ and FTO/GRA/WO_3_ electrodes.

The cycling stability and capacitance retention of the composite electrode were evaluated to assess its long-term electrochemical performance. Using 1 M LiClO_4_/PC as the electrolyte, 1000 consecutive charge–discharge cycles were carried out, as shown in Figure 12. The galvanostatic charge–discharge (GCD) tests were performed within a potential window of −0.9 to 0.0 V at a current density of 0.3 A g^–1^.

**Figure 12:**
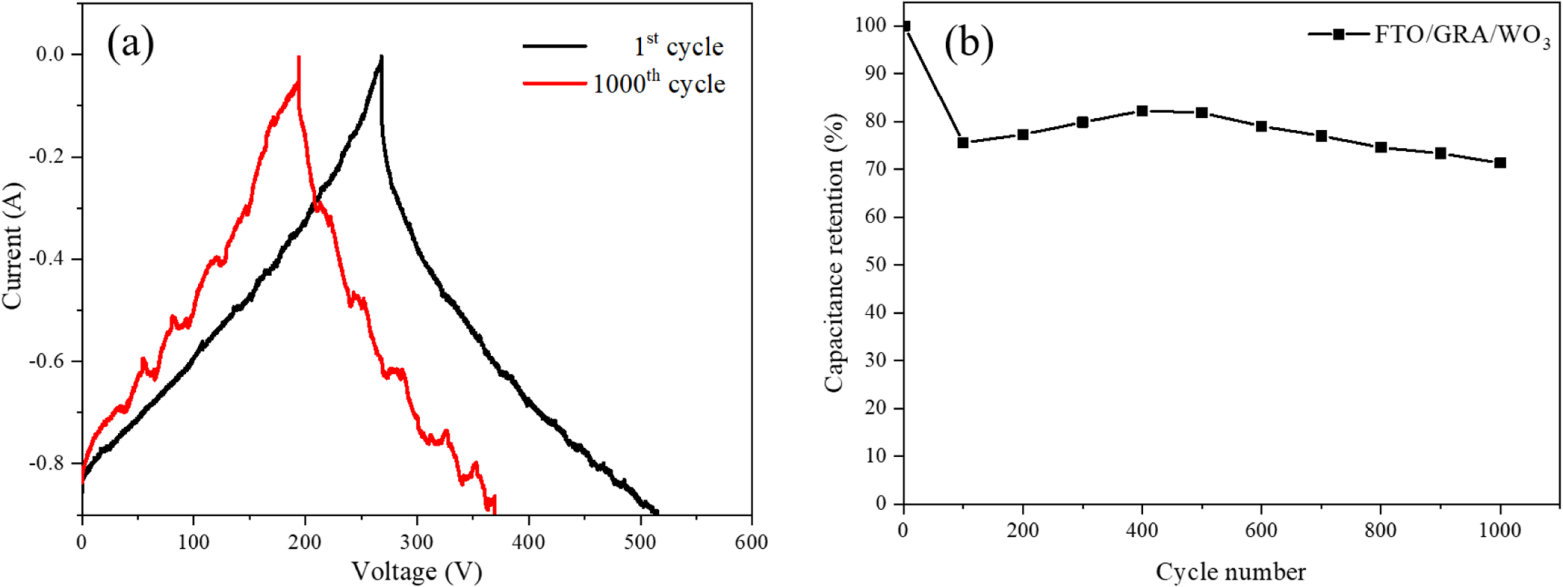
(a) GCD curve of FTO/GRA/WO_3_ at a constant current density of 0.3 A g–^1^. (b) Specific capacitance stability of the FTO/GRA/WO_3_ electrode under 1000 cycles at a constant current density of 0.3 A g^–1^.

A noticeable capacitance drop was observed during the initial 100 cycles, which can be attributed to irreversible chemical reactions occurring during the electrode activation process, resulting in partial surface oxidation and structural loss.^44^ After approximately 100 cycles, the charge–discharge behavior gradually stabilized, indicating improved reversibility of the redox reactions. After 1000 cycles, the electrode retained 71.34% of its initial capacitance, demonstrating reasonable cycling stability.

The observed decline in capacitance can be associated with the liquid electrolyte environment, where repeated lithium-ion intercalation and deintercalation during cycling may induce structural deformation or microcracks in the electrode film, disrupting conductive pathways and leading to capacity fading. In practical applications, supercapacitors typically employ solid- or gel-based electrolytes, which are expected to mitigate such degradation, thereby offering improved cycling stability, enhanced safety, and greater overall device reliability. Future work may focus on optimizing electrode–electrolyte interfaces to further enhance long-term cycling durability. While WO_3_–graphene systems show promise, the main issues revolve around the limited conductivity, insufficient electrochemical sites, mechanical instability, and poor ion diffusion in WO_3_, which reduce the supercapacitor’s performance.

## 4 Conclusions

In this work, GRA/WO_3_ composite electrodes were successfully fabricated on FTO glass substrates via a two-step ultrasonic spray deposition process. Owing to the high controllability of the ultrasonic spray technique, key parameters such as spray rate, nozzle-to-substrate distance, and the number of spray cycles were precisely adjusted to optimize film thickness and uniformity. Through systematic evaluation of electrodes prepared under different spraying conditions using cyclic voltammetry (CV) and galvanostatic charge–discharge (GCD) analyses, the optimal configuration was determined to consist of 16 graphene layers and 4 mesoporous WO_3_ layers. The optimized composite electrode exhibited a specific capacitance of 161.68 F g^–1^ at a scan rate of 5 mV s^–1^ in CV testing—significantly higher than that of the pure mesoporous WO_3_ electrode (142.18 F g^–1^). In GCD measurements at a current density of 0.5 A g^–1^, the specific capacitance further reached 235.42 F g^–1^, surpassing that of pure WO_3_ (202.35 F g^–1^). After 1000 continuous charge–discharge cycles, the electrode retained 71% of its initial capacitance, indicating satisfactory cycling stability. The introduction of a graphene interlayer between the mesoporous WO_3_ film and the FTO substrate effectively enhanced the electron transport and ion diffusion, thereby improving the overall electrochemical performance. These results demonstrate that graphene/tungsten oxide composite electrodes fabricated via the sol–gel-assisted ultrasonic spray method hold great potential as high-performance and environmentally friendly electrode materials for next-generation supercapacitors and green energy storage applications.
